# Supplementary material for: Androgens show sex-dependent differences in myelination in immune and non-immune murine models of CNS demyelination
Source: Nat Commun. 2023 Mar 22;14:1592. doi: 10.1038/s41467-023-36846-w (PMC10033728; doi:10.1038/s41467-023-36846-w)
Supplement: Supplementary file 1 — Supplementary information [file 41467_2023_36846_MOESM1_ESM.pdf]

Supplementary information for "Androgens show sex-dependent differences in myelination in immune and non-immune murine models of CNS demyelination"

Amina Zahaf[1], Abdelmoumen Kassoussi[1], Tom Hutteau-Hamel[2], Amine Mellouk[2], Corentine Marie[3], Lida Zoupi[4], Foteini Tsouki[4], Claudia Mattern[5], Pierre Bobé[2], Michael Schumacher[1], Anna Williams[4], Carlos Parras[3], Elisabeth Traiffort[1,*]

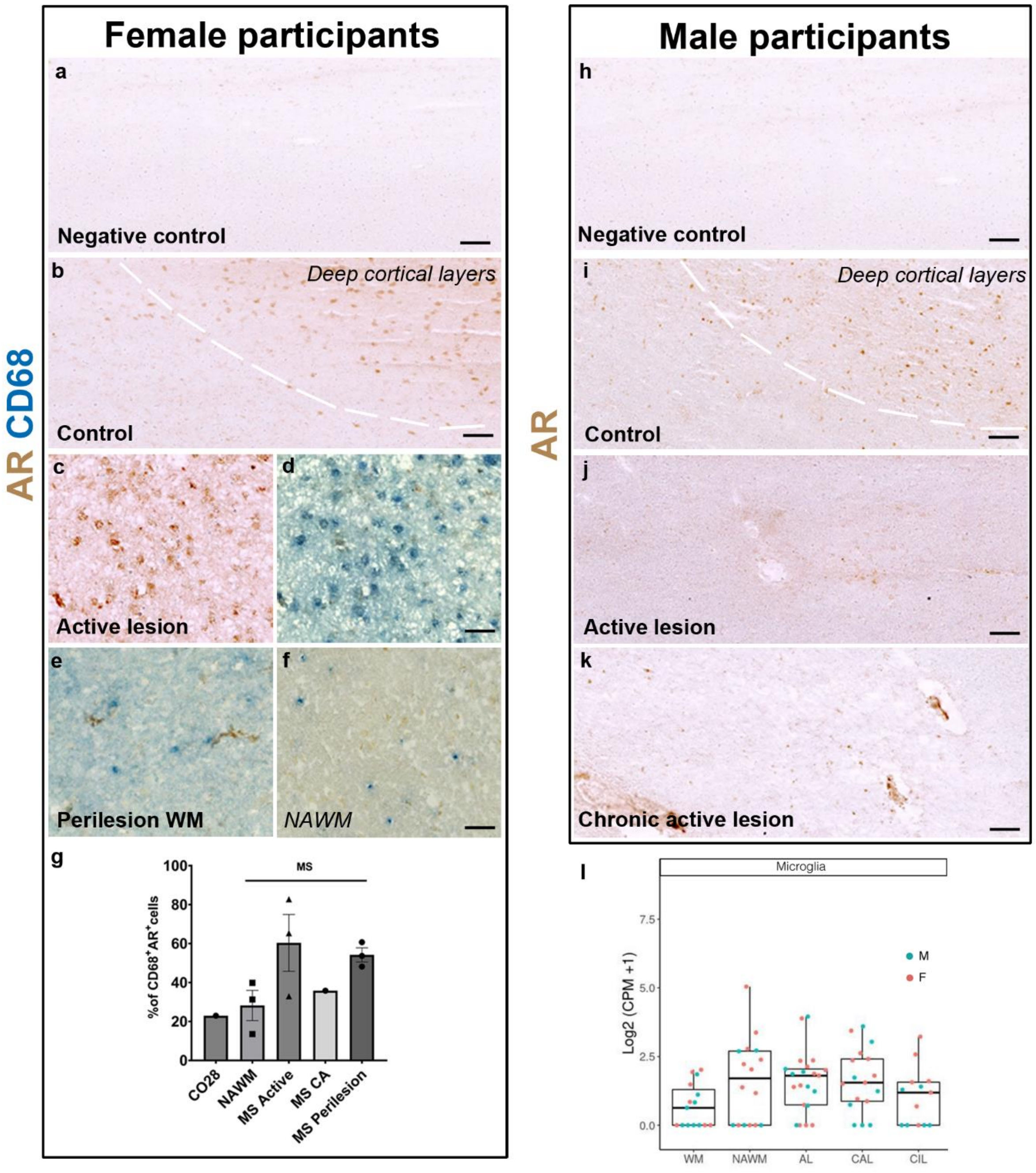

Supplementary Figure 1. AR expression in the human brain. Brain tissue (fresh frozen 10 µm sections) from women in (**a-g**) and men in (**h-k**) immunostained with an antibody directed against AR or the secondary antibody. AR immunostaining of sections encompassing the deep

cortical layers (grey matter to right of dashed line) and the subcortical white matter (left of dashed line) from healthy woman in (**b**) and man in (**i**) shows clear signal in the grey matter and lower signal in the white matter contrasting with virtually undetectable signal observed in sections incubated with only the secondary antibody in (**a, h**). An active MS lesion derived from a female patient display a strong immunostaining in (**c**) whereas an active lesion in (**j**) or a chronic active in (**k**) lesion from male patients displays an extremely faint signal. AR-expressing cells in the female active lesion co-express the CD68 marker of microglia/macrophages (blue and brown) in (**d**). **e, f** AR^+ $CD68^+$ ramified cells in the perilesion white matter in (**e**) and absence of AR-expressing cells in the normal appearing white matter (NAWM) in (**f**). **g** Percentage of $CD68^+$ AR^+ cells in the indicated brain areas from 3 female MS patients and 1 control female donor. The numbers are limited due to discontinuation of the antibody, but the percentage of $CD68^+$ AR^+ cells appear higher in active lesions and in the perilesion in (**g,** values are means ± SEM). MS CA, MS chronic active lesion. Scale bars (μm) : 100 in (**a, b, h, i, j, k**), 50 in (**c-f**). **l** AR mRNA expression in microglia from MS and control female (F, pink) or male (M, blue) donors from a publicly available independent single-nuclei RNA sequencing database (https://malhotralab.shinyapps.io/MS_broad/) seems to show higher expression in MS and female samples[1]. AL, active lesion (samples from 8M, 13F); CAL, chronic active lesion (samples from 8M, 9F); CIL, chronic inactive lesion (samples from 6M, 7F); NAWM, normal-appearing white matter (samples from 5M, 11F); WM, white matter from control donors (samples from 6M, 7F). The median is shown with box indicating 25% quartile to 75% quartile and whiskers indicating interquartile range multiplied by 1.5.

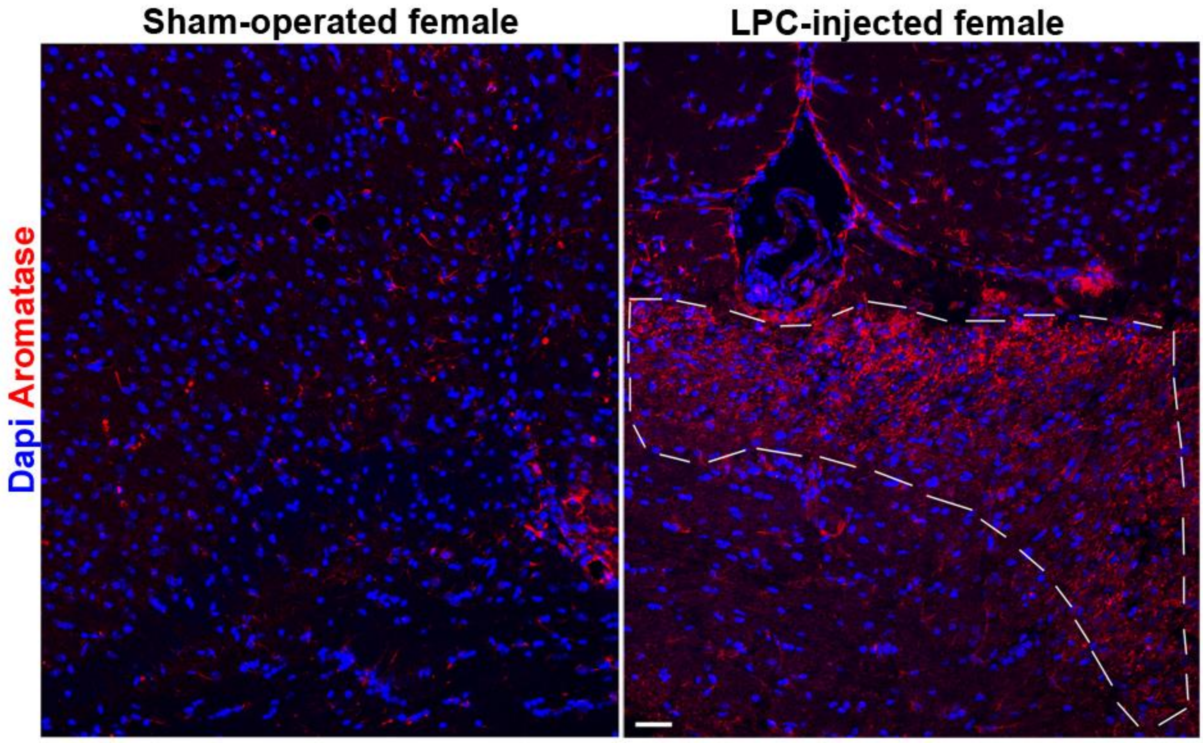

Supplementary Figure 2. Aromatase is expressed in the demyelinated corpus callosum from female mice. Aromatase immunostaining at 7 days after stereotaxic injection of LPC-vehicle (left) or LPC into the corpus callosum of female mice give rise to a faint or very strong labeling, respectively. These micrographs are representative of the labeling obtained in two

independent experiments. The LPC-induced lesion is delineated by the dashed line. Scale bar: 50 µm.

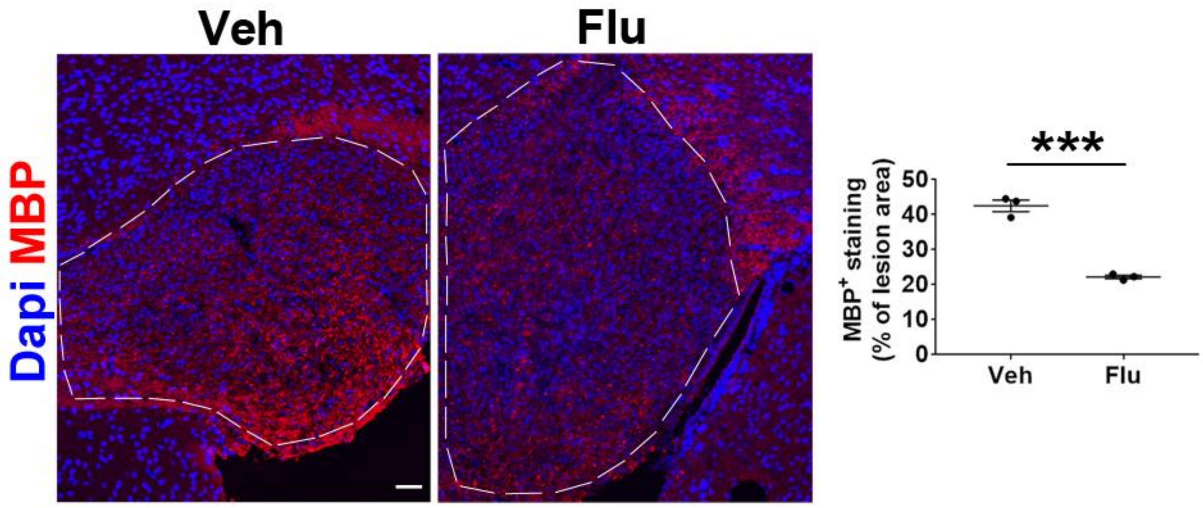

Supplementary Figure 3. Blockade of the androgen receptor by its pharmacological antagonist flutamide impedes MBP expression at 10 days after LPC injection in the 129X1/SvJ strain. Visualization and quantification of MBP immunostaining at 10 dpl. The lesions are delineated by the dashed lines. Scale bar: 50 µm. Data are presented as mean values ± SEM from n=3 animals / condition examined in a single experiment. The *p* value was calculated by using the unpaired two-tailed t-test. ***, p=0.0003. Source data are provided as a Source Data file.

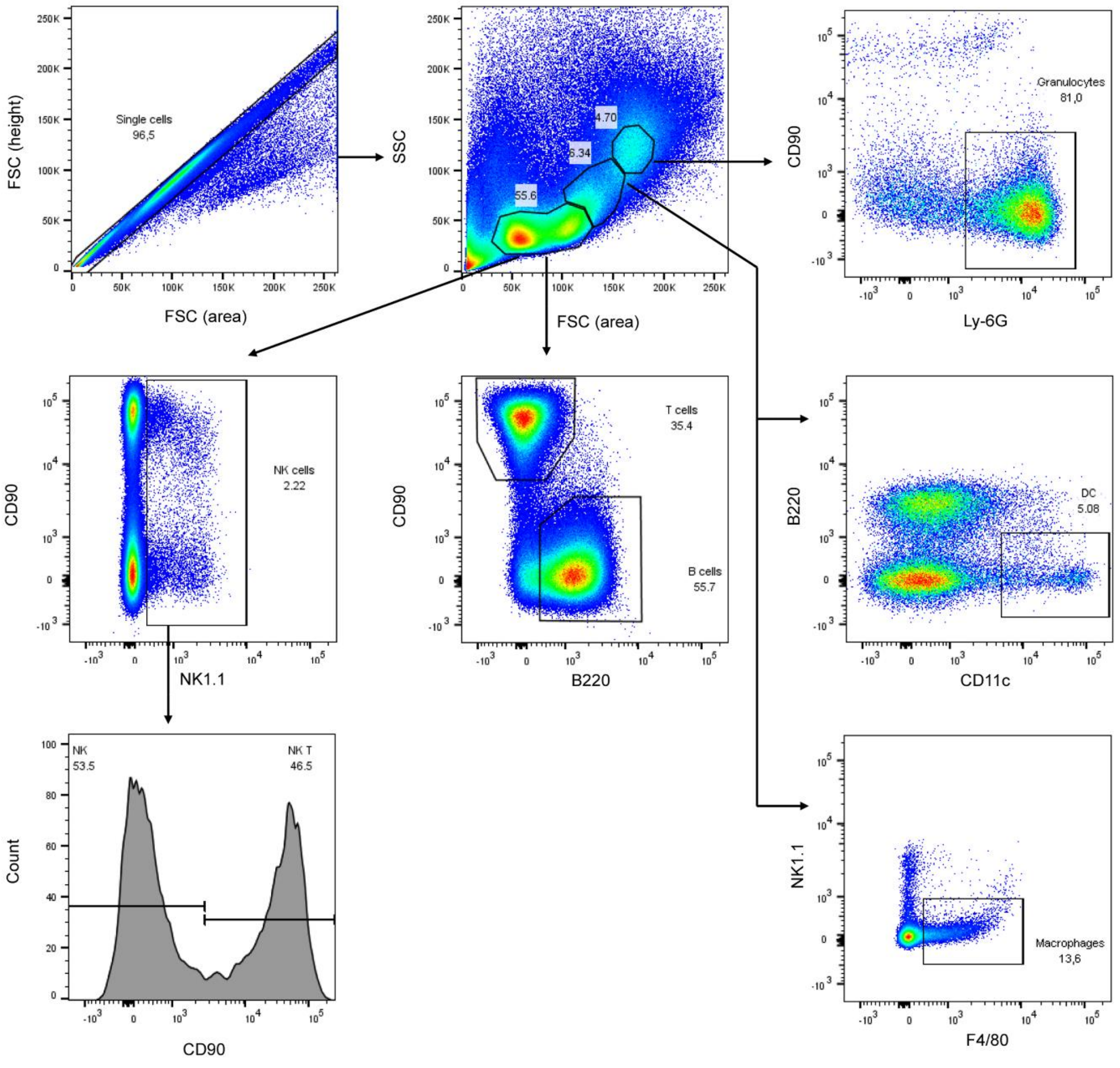

Main immune cells in spleen / lymph nodes

Supplementary Figure 4. Representative dot plot showing sequential gating for identifying the main immune cell populations in the spleen and lymph nodes. FSC and SSC parameters were used to select single, viable cells and exclude debris, dead cells and doublets. CD90 vs. B220 dot plot was then used to select B220–CD90– non-T non-B cells and B220–CD90+ T cells. The B220–CD90– non-T non-B cells were then gated by the expression of Ly6G (granulocytes), CD11c (dendritic cells), F4/80 (macrophages), NK1.1 (Natural Killer cells).

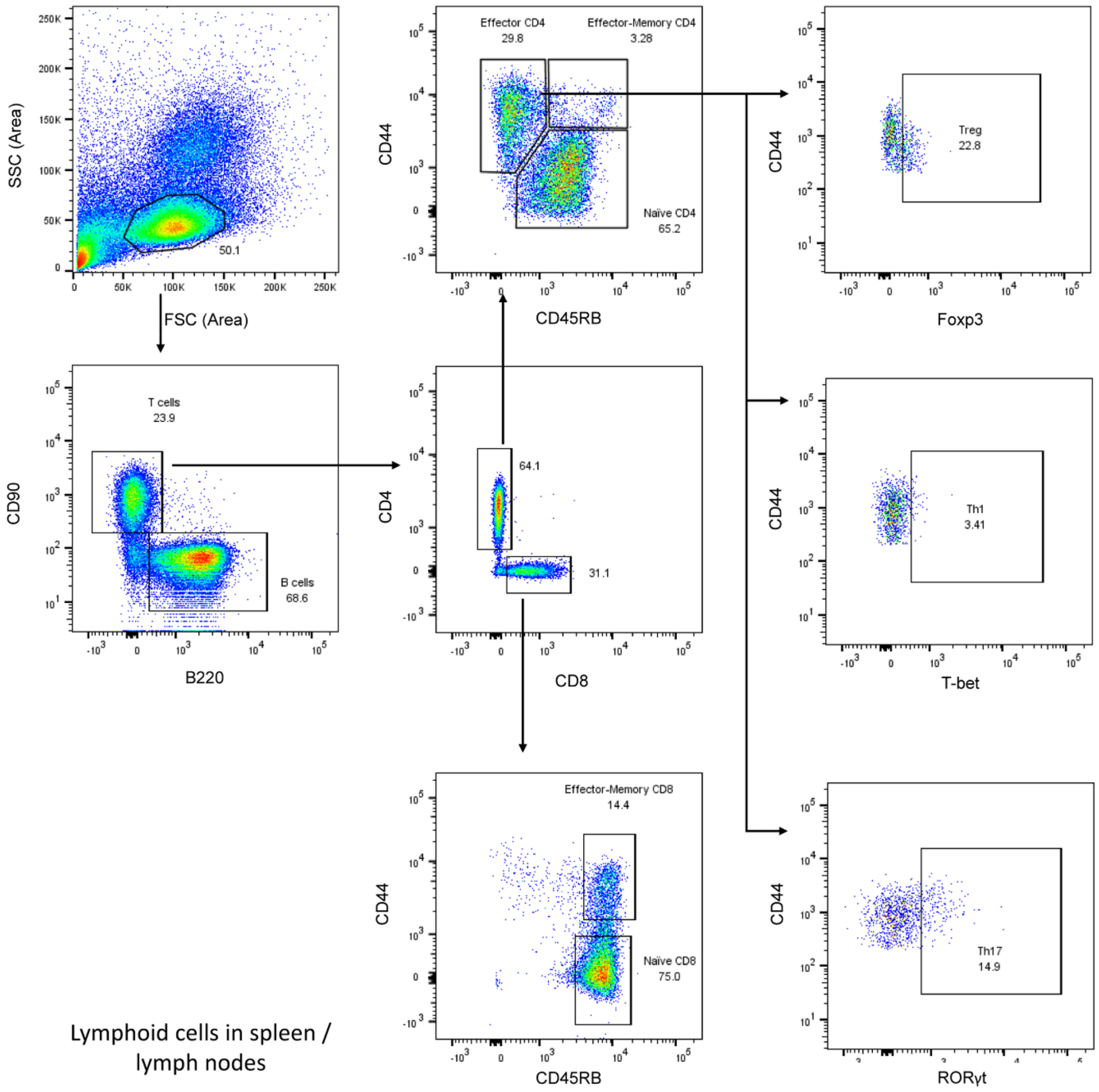

Supplementary Figure 5. Representative dot plot showing sequential gating for identifying the lymphoid cell populations in the spleen and lymph nodes. The CD90+B220– T cells were gated by the expression of CD4. The relative expression of CD44 and CD45RB was analyzed on the CD4+ T-cell subset to identify CD44loCD45RBhi naïve and CD44hiCD45RBlo effector/memory cells. Finally, the percentages of cells expressing Foxp3, T-bet and RoRγt were gated on CD44hiCD45RBlo effector/memory CD4+ T cells.

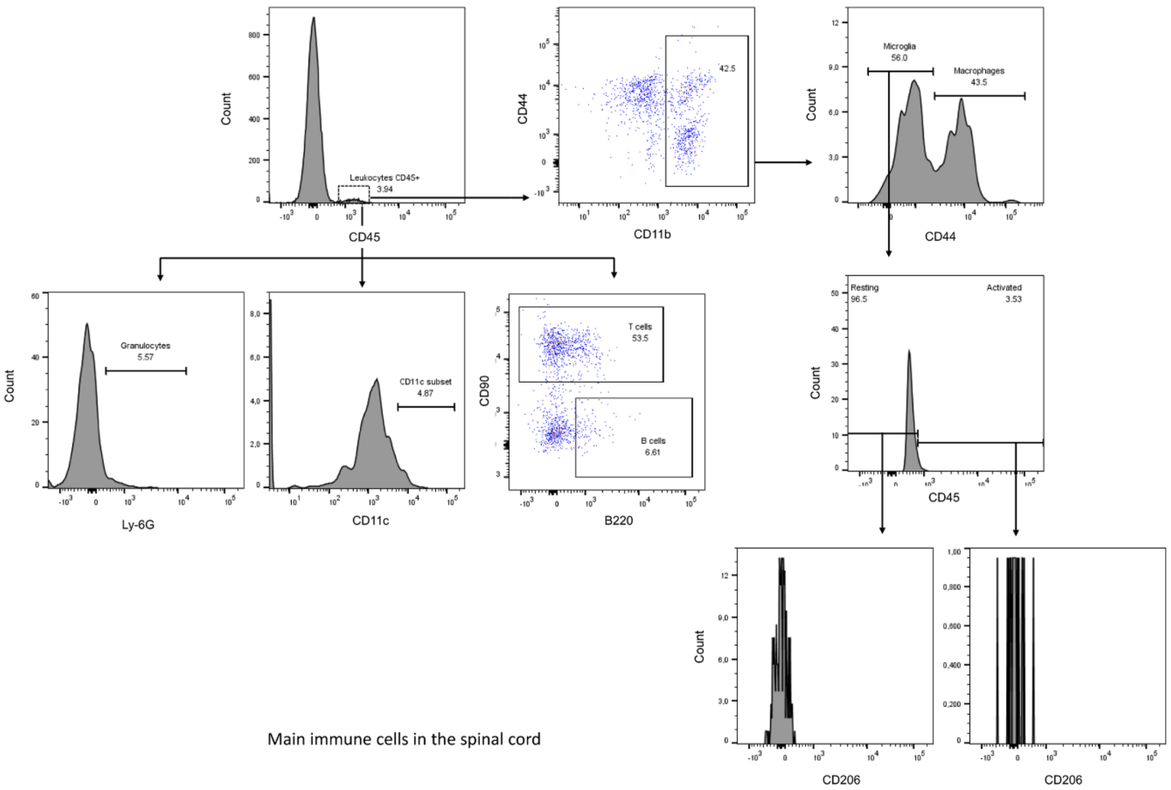

Supplementary Figure 6. Representative dot plot showing sequential gating for identifying the main immune cell populations in the spinal cord. CD45 was used to select leukocytes gated by the expression of Ly6G (granulocytes), CD11c (dendritic cells), CD90 (T cells), B220 (B cells) and CD11b (microglia/macrophages). CD44 was used for discriminating macrophages (CD44+) and microglia (CD44-). The level of CD45 expression allowed to distinguish resting (CD45 low) and activate (CD45 high) microglia. Finally, CD206 led to discriminate pro-inflammatory (CD206-) and anti-inflammatory (CD206+) microglia.

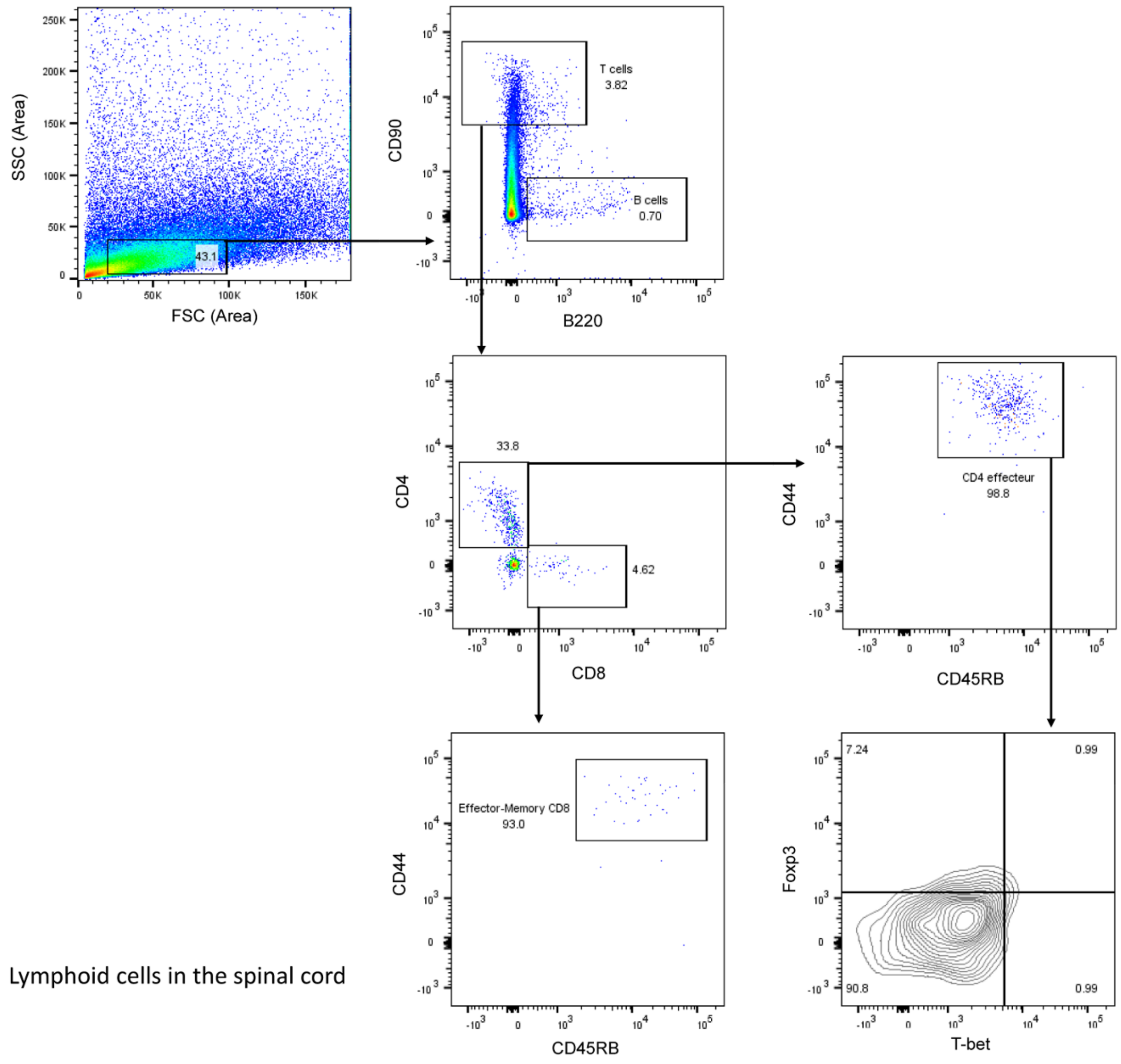

Supplementary Figure 7. Representative dot plot showing sequential gating for identifying the lymphoid cell populations in the spinal cord. The CD90+B220– T cells were gated by the expression of CD4. The relative expression of CD44 and CD45RB was analyzed on the CD4+ and CD8+ T-cell subset to identify CD44hiCD45RBlo effector/memory cells. Finally, the percentages of cells expressing Foxp3 and T-bet were gated on CD44hiCD45RBlo effector/memory CD4+ T cells.

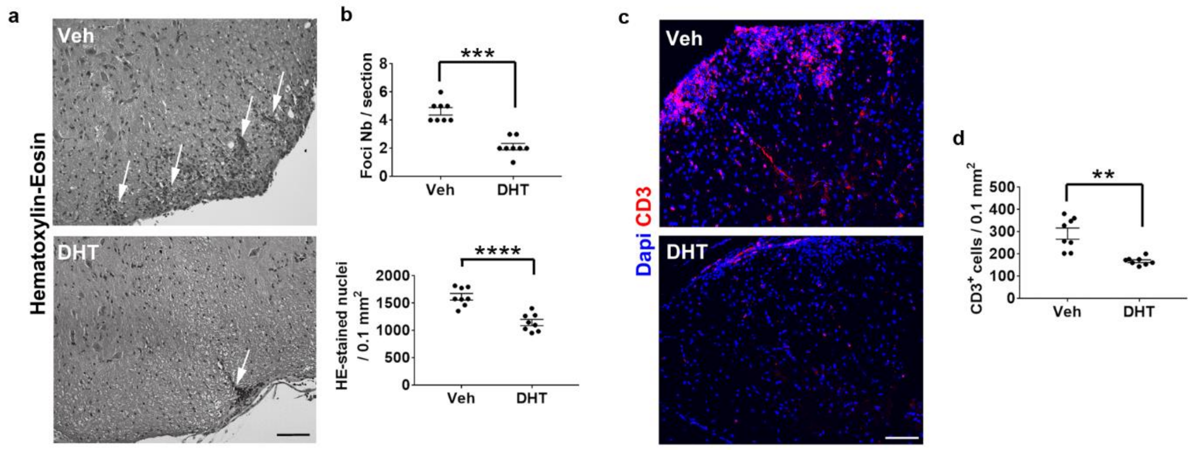

Supplementary Figure 8. DHT decreases immune cell infiltration into the spinal cord from EAE females. (a,b) Analysis of hematoxylin/eosin staining shows the higher number of foci (white arrows) and cell nuclei present in the periphery of the spinal cord from the vehicle- compared to the DHT-treated animals at 14 dpl. (c, d) Visualization and quantification of infiltrated $CD3^+$ T cell foci in each condition. Scale bars (µm): 200. Data are presented as mean values ± SEM from n=8 animals / group examined in two independent labeling experiments. *P* values were calculated by using the unpaired two-tailed t-test (**b** bottom, $p<0.0001$; **d**, $p=0.0015$) or two-tailed Mann-Whitney test (**b** top, $p=0.0002$) according to the value derived from the test for normality. Welch's correction was used for (**d**). Source data are provided as a Source Data file.

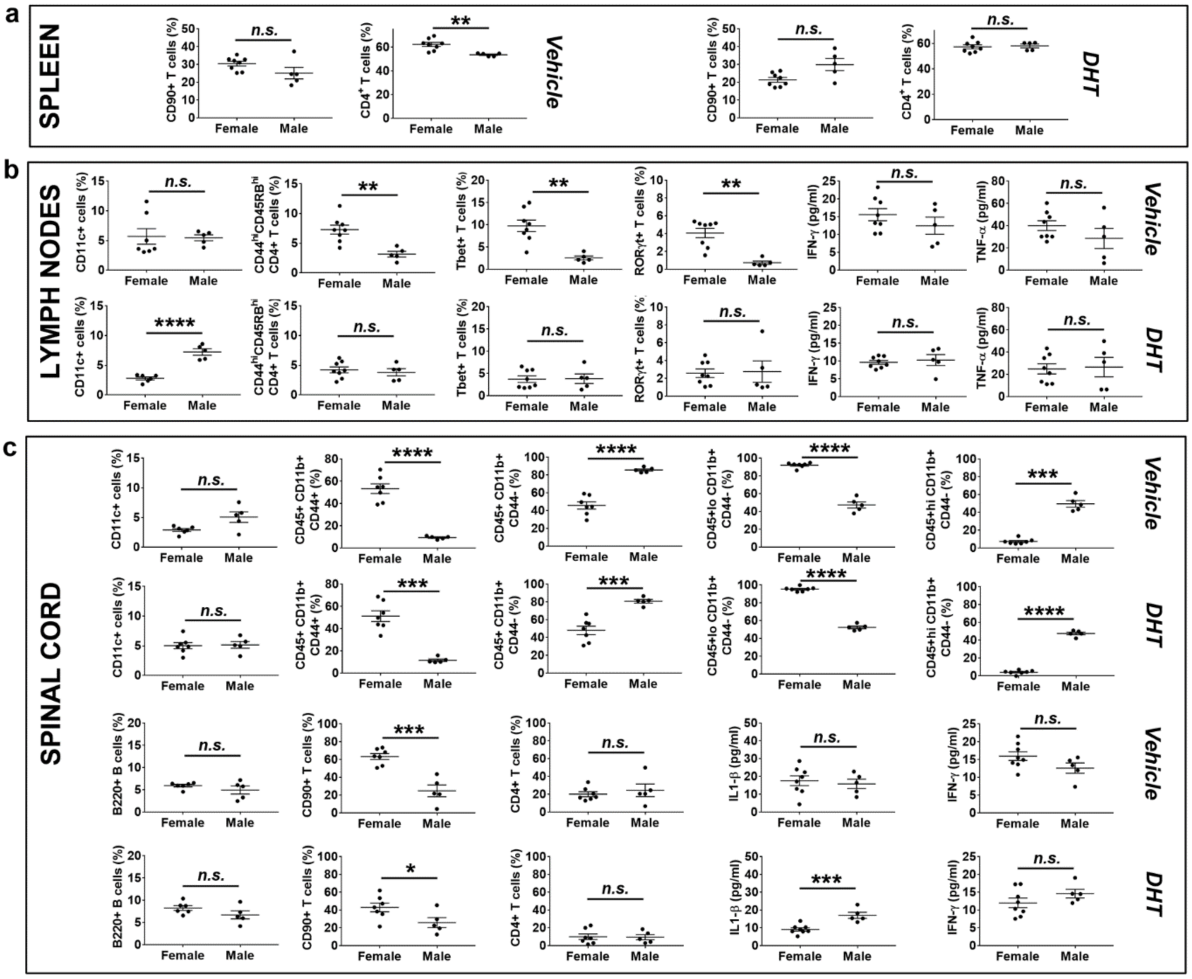

Supplementary Figure 9. Comparison of immune cell proportions present in EAE females and males under Vehicle or DHT treatment at 14 dpi. The data are derived from flow cytometry analyses performed in the spleen (**a**; n=8 females, 5 males), lymph nodes and spinal cord (**b** and **c**, respectively; n=7 females, 5 males) examined in a single experiment and shown in Figure 8. Data are presented as mean values ± SEM. *P* values were calculated by using the unpaired two-tailed t-test or Mann-Whitney test according to the value derived from the test for normality. Whelch's correction was used in (**c**). *, p≤0.05; **, p≤0.01; ***, p≤0.001; ****, p≤0.0001. n.s., non-significant. Source data are provided as a Source Data file.

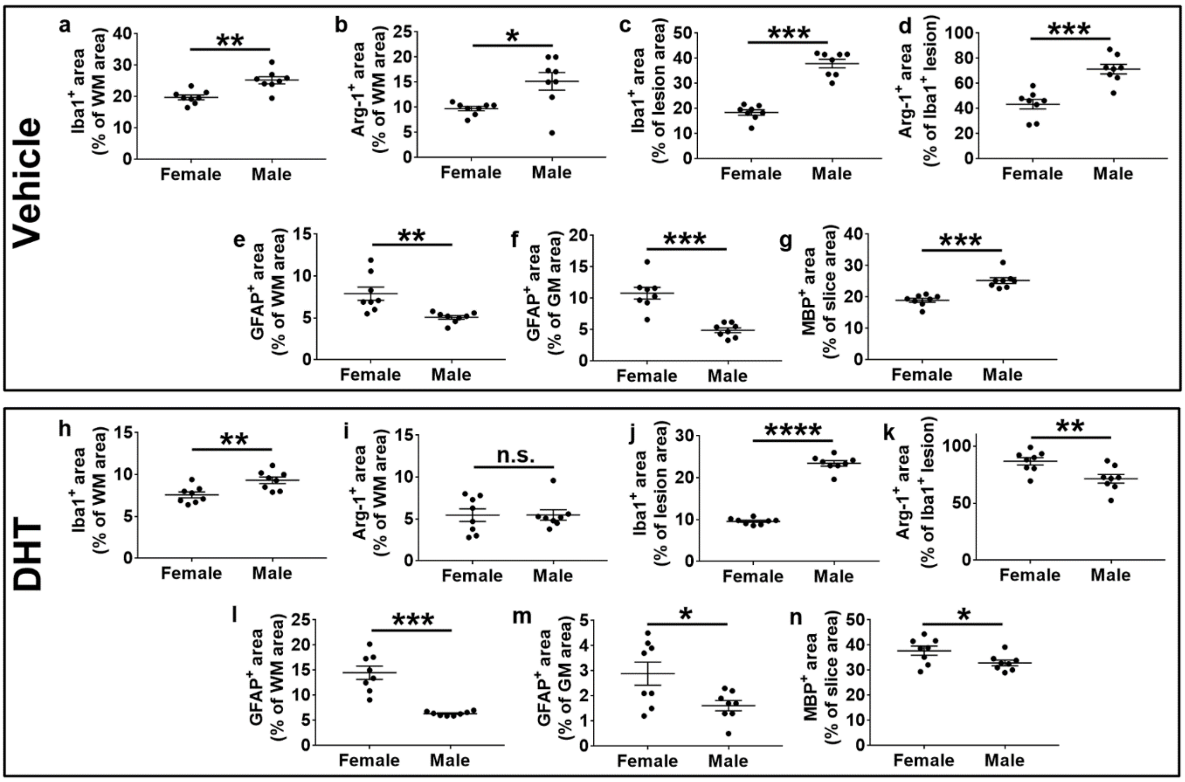

Supplementary Figure 10. Comparison of parenchymal inflammatory cells and levels of myelin expression in EAE females and males under Vehicle (a-g) or DHT (h-n) treatment at 14 dpi. The data are derived from spinal cord immunostaining experiments using the microglial (Iba1, Arg-1), astroglial (GFAP) and myelin (MBP) markers shown in Figure 9. Data are presented as mean values ± SEM from n=8 animals / group examined in a single experiment. *P* values were calculated by using the unpaired two-tailed t-test or Mann-Whitney test according to the value derived from the test for normality. Welch's correction was used for (**b, e, f, j, l, m**). *, p≤0.05; **, p≤0.01; ***, p≤0.001; ****, p≤0.0001. n.s., non-significant. Source data are provided as a Source Data file.

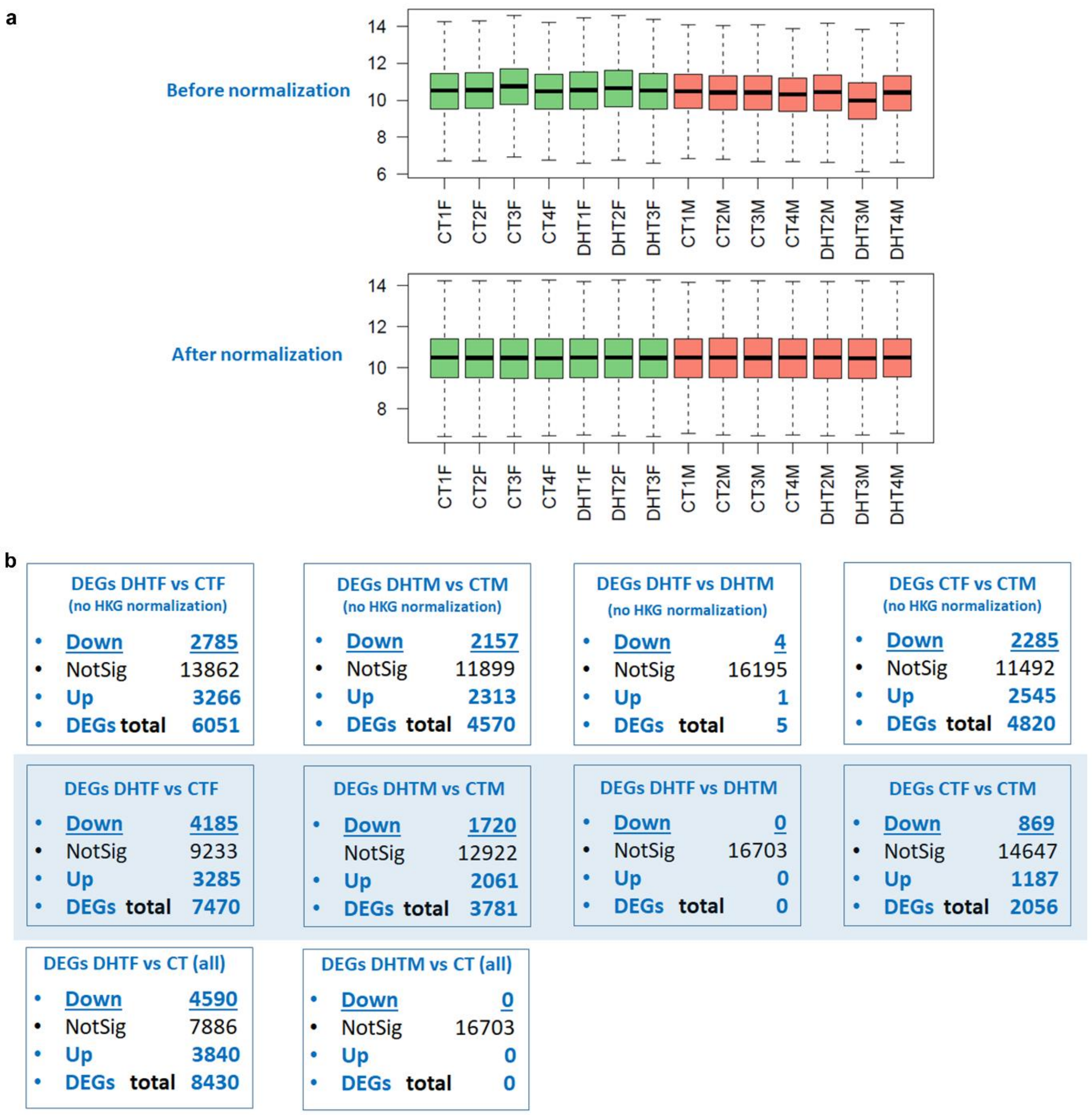

Supplementary Figure 11. Normalization of female and male RNA-Seq analysis. (a) Integration of female and male datasets by using normalization via housekeeping genes present in the two datasets aimed at reducing unwanted variation from RNA-Seq data (RUVSeq). (b) Differential analysis performed before (top) or after (bottom) housekeeping gene normalization indicating comparable numbers of DEGs in particular between females (n=3 / group) treated (DHTF) or not (CTF) with DHT, and between males (n=3 / group) treated (DHTM) or not (CTM) with DHT examined in two independent experiments. CT corresponds to non-treated male and female animals. In order to control the false discovery rate (FDR, p-adjust), multiple testing correction was performed by using the Benjamini-Hochberg method.

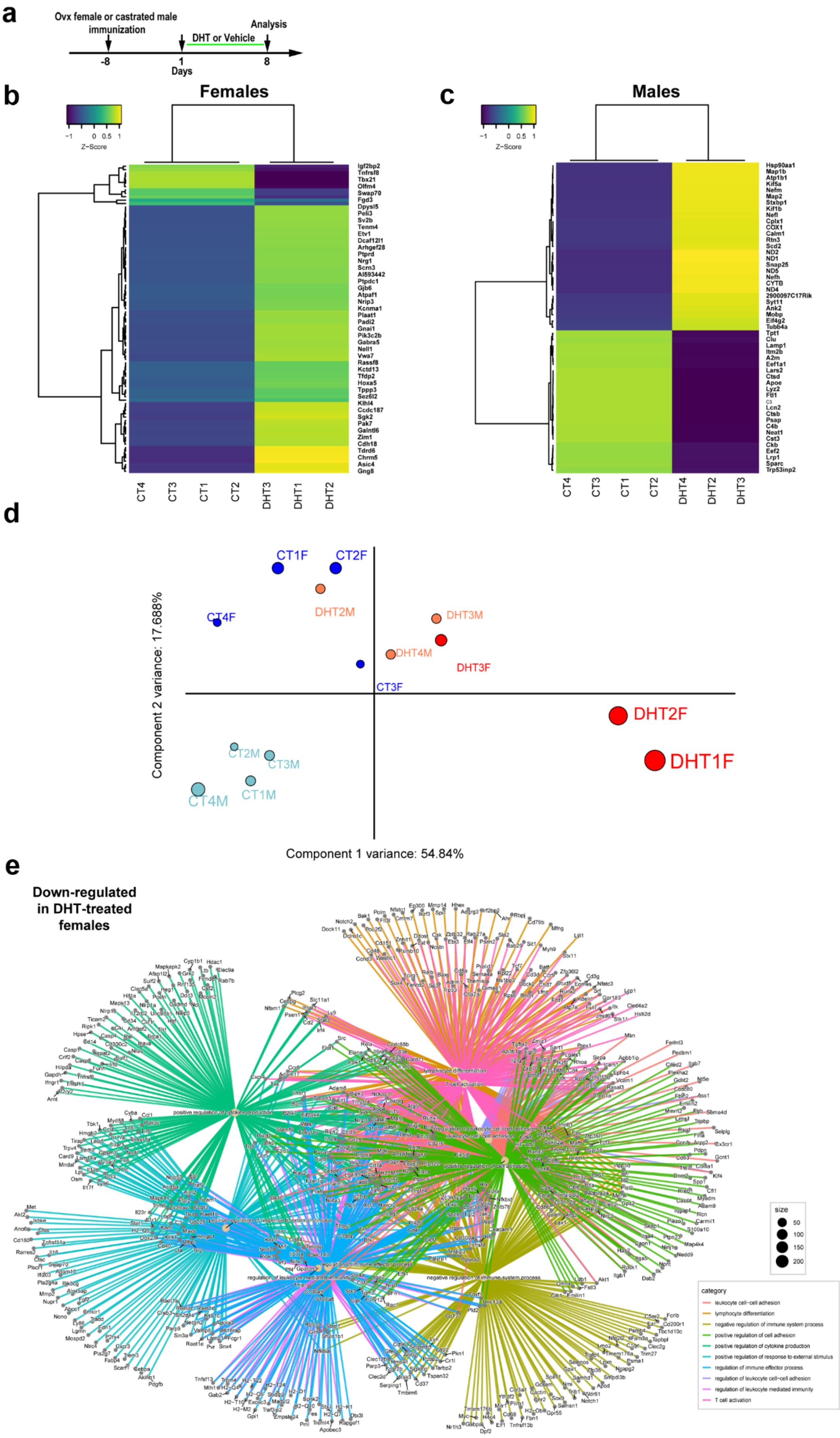
a
Ovx female or castrated male immunization
Analysis
DHT or Vehicle
-8
1
8
Days
b
Females
-1 0 0.5 1
Z-Score
Igf2bp2
Tnfrsf8
Tbx21
Olfm4
Swap70
Fgd3
Dpysl5
Peli3
Sv2b
Tenm4
Etv1
Dcaf12l1
Arhgef28
Ptprd
Nrg1
Scrn3
AI593442
Ptpdc1
Gjb6
Atpaf1
Nrip3
Kcnma1
Plaat1
Padi2
Gnai1
Pik3c2b
Gabra5
Nell1
Vwa7
Rassf8
Kctd13
Tfdp2
Hoxa5
Tppp3
Sez6l2
Klhl4
Ccdc187
Sgk2
Pak7
Galnt6
Zim1
Cdh18
Tdrd6
Chrm5
Asic4
Gng8
CT4
CT3
CT1
CT2
DHT3
DHT1
DHT2
c
Males
-1 0 0.5 1
Z-Score
Hsp90aa1
Map1b
Atp1b1
Kif5a
Nefm
Map2
Stxbp1
Kif1b
Nefl
Cplx1
COX1
Calm1
Rtn3
Scd2
ND2
ND1
Snap25
ND5
Nefh
CYTB
ND4
2900097C17Rik
Syt11
Ank2
Mobp
Eif4g2
Tubb4a
Tpt1
Clu
Lamp1
Itm2b
A2m
Eef1a1
Lars2
Ctsd
Apoe
Lyz2
Ftl1
C3
Lcn2
Ctsb
Psap
C4b
Neat1
Cst3
Ckb
Eef2
Lrp1
Sparc
Trp53inp2
CT4
CT3
CT1
CT2
DHT4
DHT2
DHT3
d
CT1F
CT2F
DHT2M
CT4F
DHT3M
DHT4M
DHT3F
CT3F
DHT2F
DHT1F
CT2M
CT3M
CT4M
CT1M
Component 2 variance: 17.688%
Component 1 variance: 54.84%
e
Down-regulated in DHT-treated females
size
50
100
150
200
category
leukocyte cell-cell adhesion
lymphocyte differentiation
negative regulation of immune system process
positive regulation of cell adhesion
positive regulation of cytokine production
positive regulation of response to external stimulus
regulation of immune effector process
regulation of leukocyte cell-cell adhesion
regulation of leukocyte mediated immunity
T cell activation

Supplementary Figure 12. RNA-Seq analysis of the spinal cord derived from EAE mice therapeutically treated with DHT reveal major differences between females and males. (a) Scheme of the experimental protocol to obtain spinal cord samples of EAE and control animals for bulk RNA-Seq analysis. (b) Heat map clustering representations of the top 50 differentially expressed genes (DEGs), showing clustering segregation of DHT-treated samples (DHT, n=3 / sex) in females (b) and males (c) samples compared to their respective controls (CT, n=4 per sex) examined in a single experiment. (d) PCA plot of two first components with their contribution to the variance of both females and males DHT-treated (DHT) and control (CT) samples after normalization by house-keeping genes (see Methods). The size of the sample name and the circle indicate the relative contribution to the total variance. (e) CnetPlot depicting the top 10 biological process and linkages of down-regulated genes in DHT-treated females compared to controls.

a

Female

	Specification	Proliferation	Migration	Survival	Differentiation	Myelination	Total
TotalScores	-0,09	-19,61	-13,79	-4,25	-3,49	20,76	-20,47
Total positive	13,15	17,09	8,03	19,25	49,30	47,08	153,91
Total negative	-13,23	-36,70	-21,83	-23,50	-52,80	-26,32	-174,38
Number genes	29	43	23	32	106	94	327
Positive genes	16	14	9	15	57	55	166
Negative genes	13	29	14	17	49	39	161

b

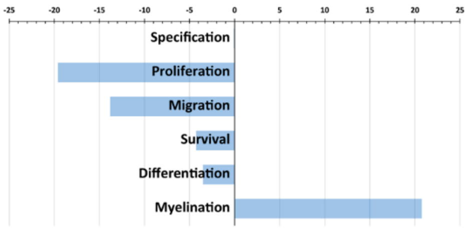

c

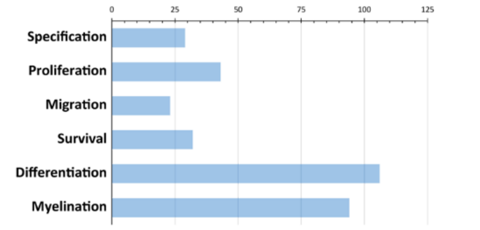

d

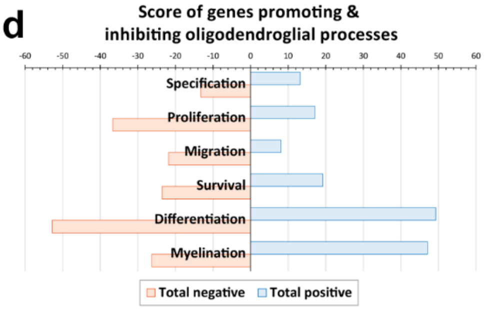

e

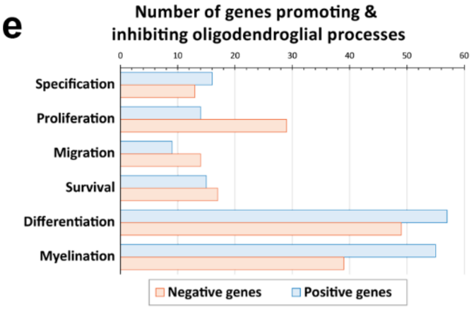

f

Male

	Specification	Proliferation	Migration	Survival	Differentiation	Myelination	Total
TotalScores	2,77	2,92	-0,35	-3,41	0,04	10,69	12,65
Total positive	6,35	5,99	2,45	3,52	17,04	23,22	58,57
Total negative	-3,59	-3,07	-2,80	-6,93	-17,00	-12,53	-45,92
Number genes	12	13	7	13	47	50	142
Positive genes	7	7	4	5	27	31	81
Negative genes	5	6	3	8	20	19	61

g

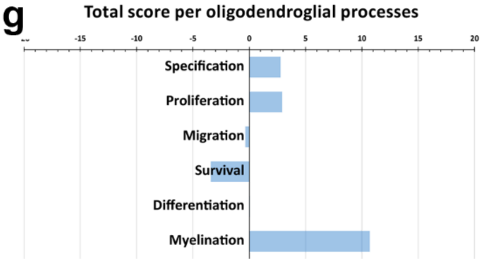

h

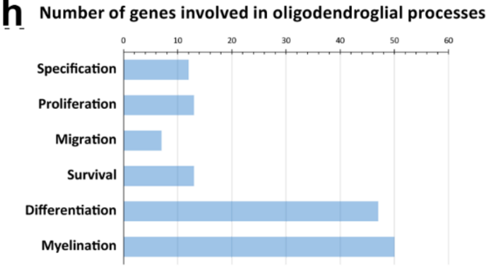

i

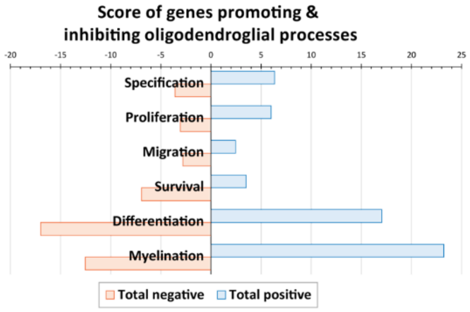

j

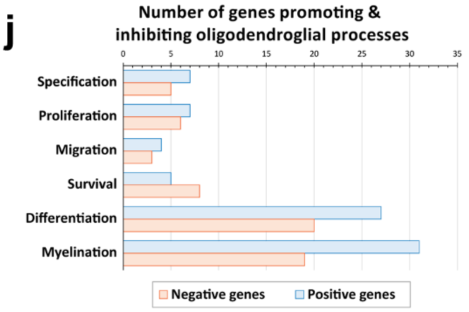

k

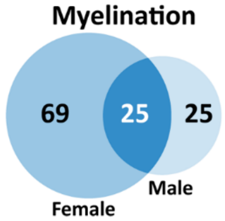

l

Common (25)	Ar	B4galt6	Cadm1	Cadm2	Ckb	Cnr1	Ctnnb1	Ctsd	Fa2h	Ilk	Mapk1	Myef2	Ncstn
	Neat1	Nf1	Nrg1	Opalin	Pak1	Pak3	Seh1l	Tfeb	Tns3	Tppp	Ugt8a	Vamp2	
Female only (69)	Adam17	Akt1	Aplp2	App	Arhgap5	Asah1	Asic1	Aspa	Ate1	Atg5	B4galt5	Bcan	Cd9
	Cdk5	Chd7	Cmtm5	Cntn1	Cntn2	Cyfip2	Ddx20	Ddx54	Dnmt1	Edn1	Eed	Fgfr2	Fth1
	Gipc1	Gjc2	Gjc3	Gpr37	Hexb	Itgb1	Jag1	Kcnj10	Kcnk3	Kif3a	Lig1	Lpar1	Marcks
	Mbp	Mog	Mtor	Myoc	Omg	Oprk1	Oprl1	Oprm1	Padi2	Parp1	Plekha1	Plp1	Ppp1r16b
	Ptprz1	Rnd2	Rras2	Skap2	Smarca2	Smurf1	Sp7	Sptbn1	St3gal2	Stat1	Suz12	Tenm4	Tgfb1
	Tlr2	Tmem106b	Vhl	Wasf1									
Male only (25)	Acox1	Ago2	Agps	Arsa	Bdnf	Bmp4	Cers2	Ckap5	Cyp51	Ermn	Galc	Gas6	Gatm
	Gdpd2	Il33	Mal	Mcoln1	Mettl14	Pde4b	Pparg	Pten	Qk	Scrib	Sirt2	Ugcg	

Supplementary Figure 13. Detailed analyses based on our curation strategy of scoring the deregulated genes implicated in the different processes of oligodendrogenesis. Tables and barplots showing the contribution of oligodendroglial curated DEGs genes to promote (positive, blue) or inhibit (negative, orange) each process of oligodendrogenesis in females (**a-e**) and in males (**f-g**). In all the bar plots, the title of the x-axis is indicated just above the axis. The title of the y-axis corresponds to the oligodendrogenesis processes labeled in each bar. Note that DHT mainly promotes myelination. (**k, l**) Venn-diagram comparing the number of oligodendroglial genes involved in the myelination process shared (25 genes) between DEGs in females (94 genes) and males (50 genes).

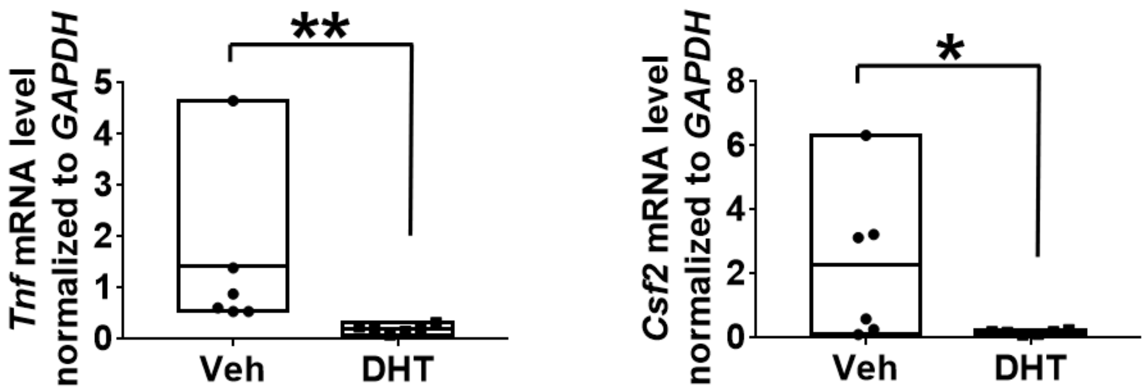

Supplementary Figure 14. Validation of the downregulation of *Tnf* and *Csf2* expression in DHT-treated EAE females. Quantitative RT-PCR was performed on spinal cord mRNA from Vehicle or DHT-treated EAE female mice (n=6 / group) analyzed at 14 dpi and examined in a single experiment. The upper, middle and lower horizontal lines of the boxplots represent the upper, median and lower quartile, respectively. *P* values were calculated by using the unpaired Mann-Whitney test. *, p=0.037; **, p=0.002. Source data are provided as a Source Data file.

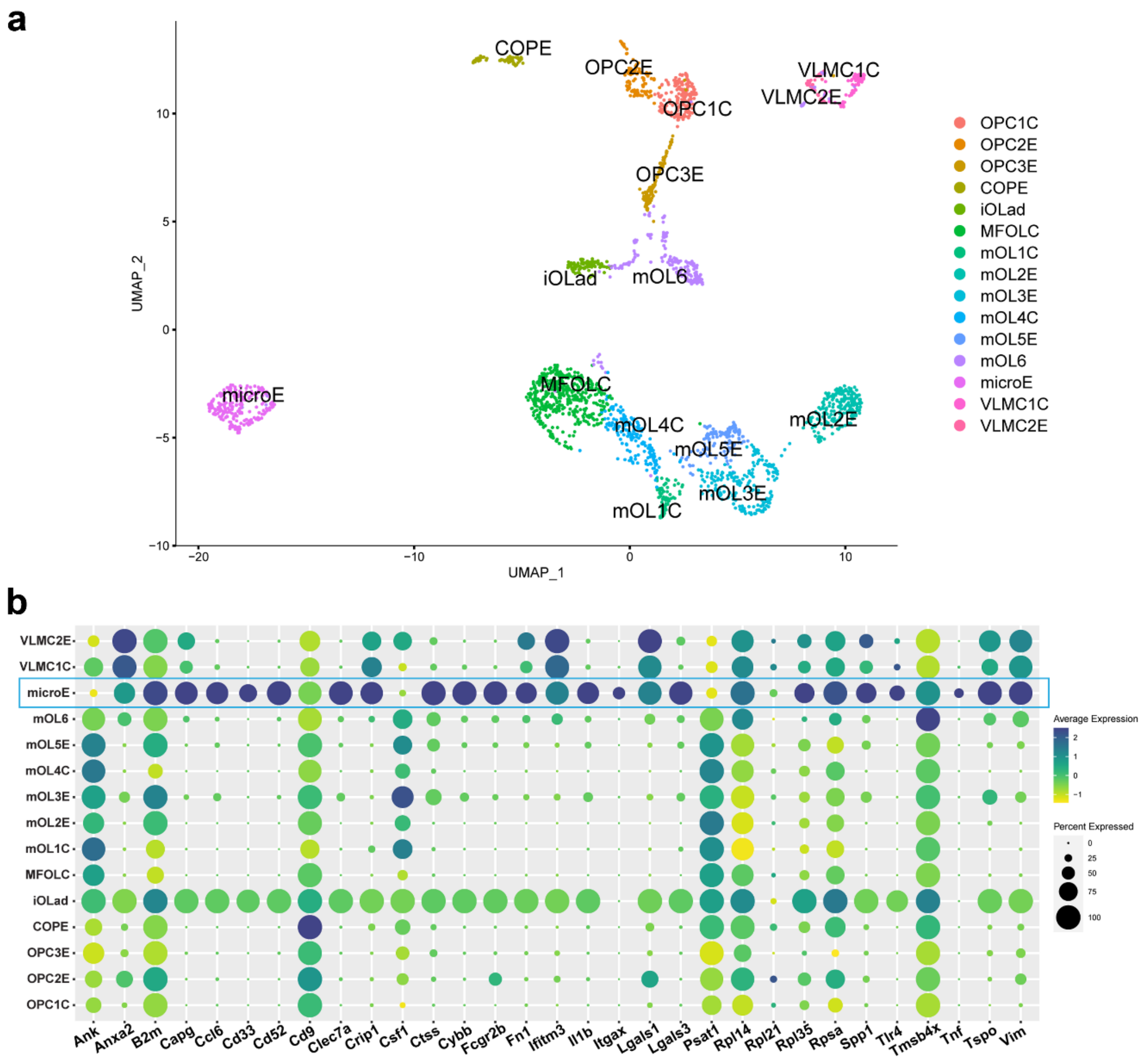

Supplementary Figure 15. Genes from prototypical classes of microglia specifically deregulated in EAE females (and not in EAE males) under DHT treatment belong to the EAE microglial cluster. The analysis has been performed by using the scRNA-Seq dataset from mouse EAE model (Falcao et al., 2018; GSE113973). **a** UMAP representation of 15 main clusters from control and EAE cells. Clusters are labelled largely following Falcao et al., 2018 nomenclature, with E-suffix standing for EAE, and C-suffix standing for Control. iOLad, adult immature oligodendrocyte. COP, committed oligodendrocyte precursors; MFOL, myelin forming oligodendrocyte; mOL, myelinating oligodendrocyte; micro, microglia; VLMC, vascular lepto-meningeal cell. (**b**) Dotplot visualization of 31 microglial genes downregulated only in DTH-treated females, showing that most genes (21 out of 31) mainly expressed in EAE microglial cells (microE cluster, blue rectangle). The color and size of each dot characterize the average and percent expression, respectively as indicated in the right scales.

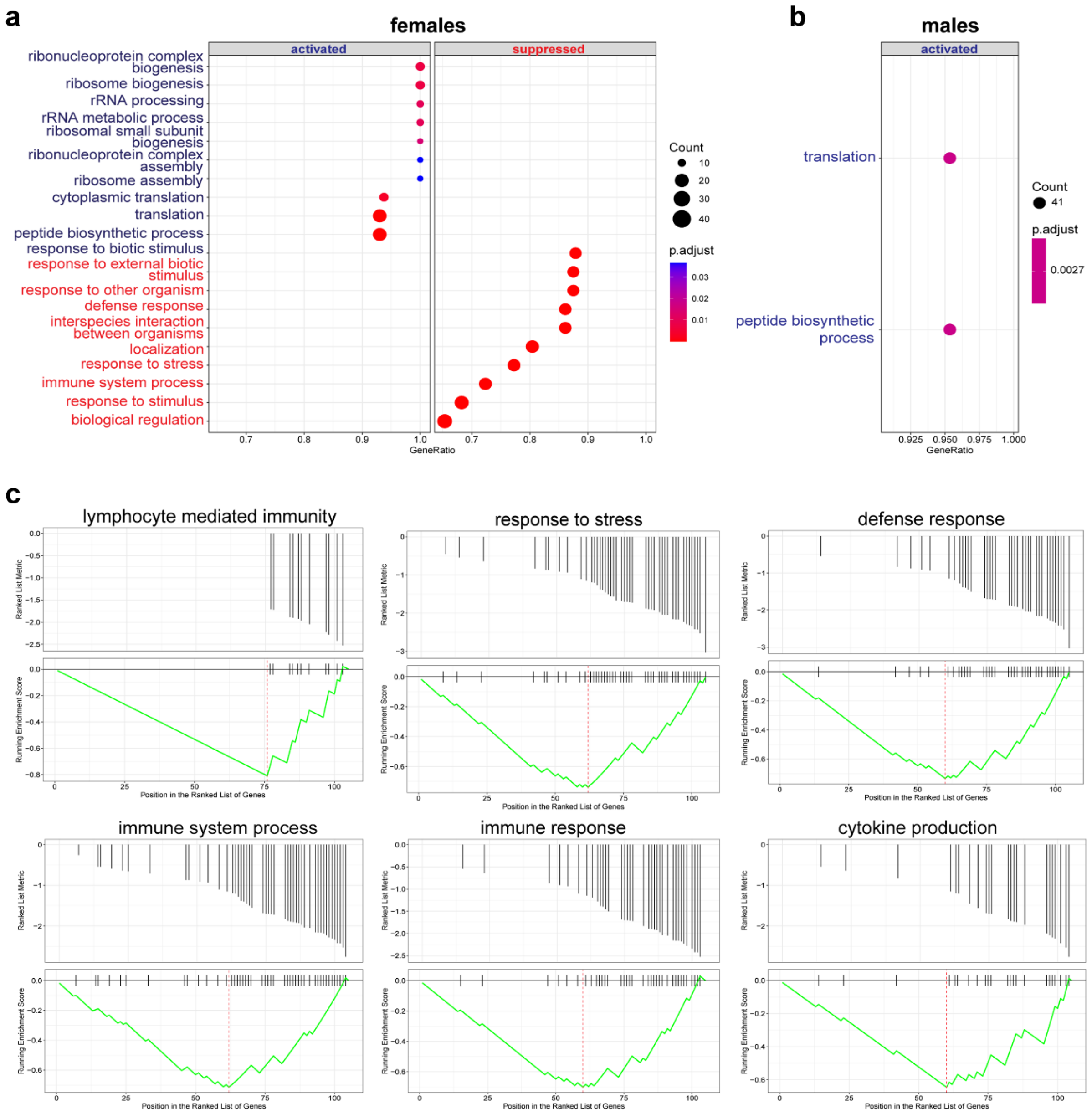

Supplementary Figure 16. Microglial genes show enrichment in gene set related to immune and inflammatory processes in DTH-treated females but not males. a, b Dotplot visualization of gene set enrichment analysis (GSEA) using microglial specific genes (Supplementary Table 12) according to their deregulation in DTH-treatment comparisons, representing the top 10 gene sets activated or suppressed in (**a**) females (DTH-treated compared to controls) and (**b**) males (DTH-treated compared to controls). Note that only in females there is an enrichment in suppressed gene sets related to immune and inflammatory processes, with no enrichment of suppressed gene sets in males. **c** GSEA plots visualization illustrating immune/inflammatory gene sets suppressed (i.e., genes being downregulated) in DTH-treated females. In order to control the false discovery rate (FDR, p-adjust), multiple testing correction was performed by using the Benjamini-Hochberg method.

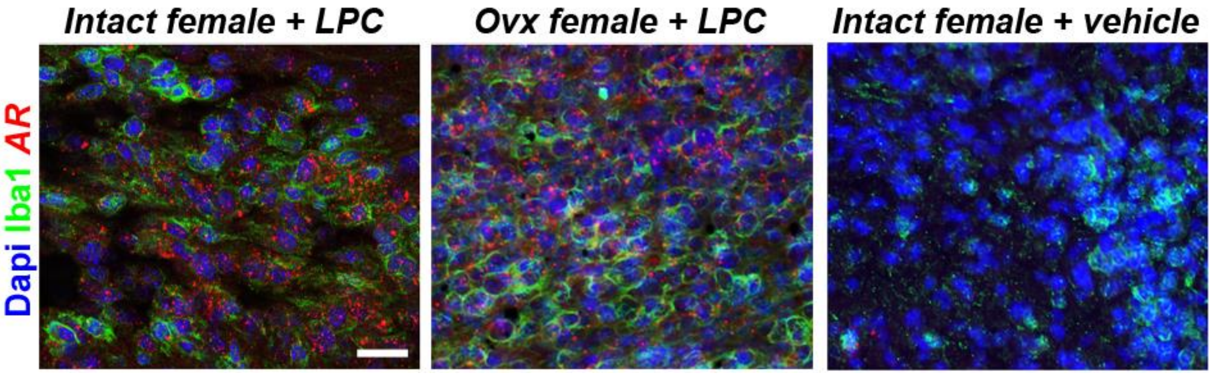

Supplementary Figure 17. ***AR*** **upregulation occurs in both intact and ovariectomized female mice.** Slices located at the level of the demyelinated area derived from intact (left) and ovariectomized (Ovx; (middle) female mice injected with LPC into the corpus callosum. In both conditions, AR^+ RNAscope signals can be detected in $Iba1^+$microglial cells. Sham animals stereotaxically injected with LPC-vehicle (right) do not display *AR* upregulation. These micrographs are representative of the labeling obtained in two independent experiments. Scale bar: 25 µm.

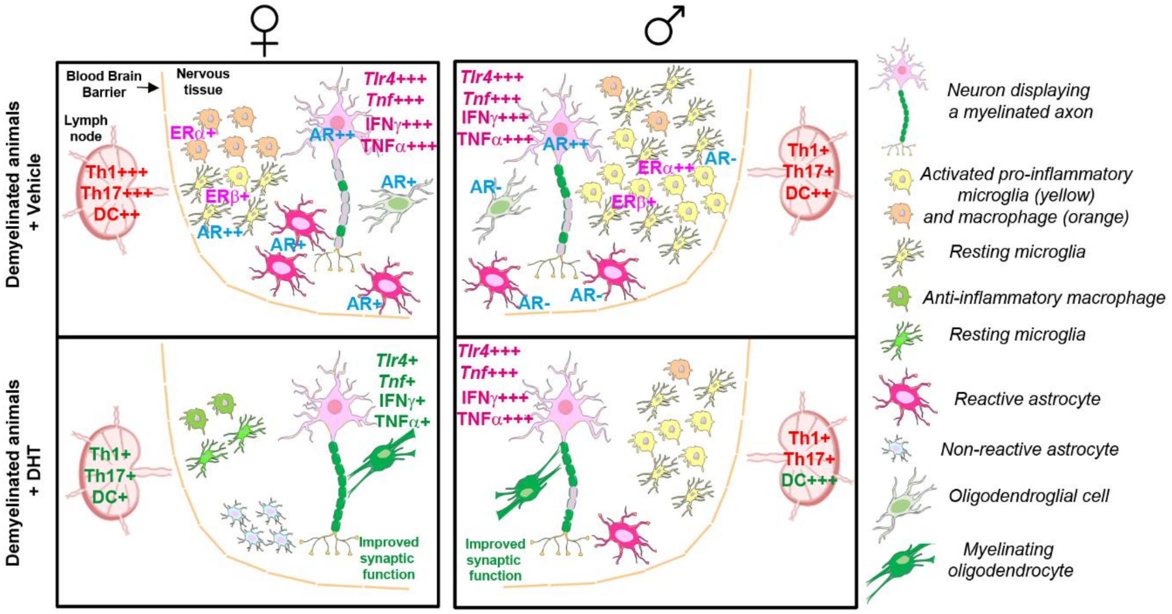

Supplementary Figure 18. Graphical abstract.

Supplementary Table 1. Characteristics of human post-mortem cases included in RNAscope analysis.

Sample ID	Age (y)	Sex	PMI (h)	Areas examined
SD008/18	69	F	94	WM
SD038/15	44	M	95	WM
SD031/14	45	F	40	WM
SD042/18	73	F	74	WM
SD012/15	45	M	51	WM
SD021/17	67	M	68	WM
CO40	61	F	NK	WM
CO41	66	M	NK	WM
CO67	67	F	32	WM
CO75	88	M	8	WM
MS98	57	M	21	AL, CAL, NAWM
MS106	39	F	18	CAL, CIL, NAWM
MS108	53	M	46	CIL, NAWM
MS200	44	F	19	AL, CAL, CIL, NAWM
MS235	53	M	14	AL, CIL, NAWM
MS242	57	F	12	AL, NAWM
MS245	64	M	25	AL, CAL, CIL, NAWM

MS249	59	F	8	AL, CIL, NAWM
MS298	72	M	11	CAL, NAWM
MS300	56	F	16	AL, CIL, NAWM
MS361	60	F	10	AL, CIL, NAWM

AL, active lesion; CAL, chronic active lesion; CIL, chronic inactive lesion; NAWM, normal-appearing white matter; PMI, post-mortem interval; other metadata are available on request.

Supplementary Table 2. Characteristics of human post-mortem cases included in immunostaining.

Sample ID	Age (years)	Sex	PMI (hours)	Number and type of lesions
CO25	35	M	22	-
CO28	60	F	13	-
CO39	82	M	21	-
MS100	46	M	7	1 AL
MS121	49	F	24	2 AL<br>1 CAL
MS136	40	M	10	2 AL<br>3 CAL
MS154	34	F	12	1 AL
MS176	37	M	12	1 CAL
MS230	42	F	31	3 AL

AL, active lesion; CAL, chronic active lesion; PMI, post-mortem interval; other metadata available on request.

Supplementary Methods

Chromogenic immunohistochemistry on human cryosections. Human tissue cryosections (10 µm) were fixed in 4% paraformaldehyde in 1xPBS for 1 hour at room temperature. Endogenous peroxidase and alkaline phosphatase activity was blocked by a 10 min incubation with Bloxall solution (SP-6000, Vector Laboratories). The sections were subsequently blocked with a ready-to-use 2.5% normal horse serum (Vector Laboratories) for least 30 min and incubated overnight with primary antibodies diluted in antibody diluent (Spring Bioscience, ADS-125) at 4°C in a humidified chamber. Primary antibodies: For AR, 3 different antibodies were tested, but only one worked - the rabbit polyclonal anti-AR/Androgen Receptor Antibody (N-20) sc-816 (Santa-Cruz Biotechnology; 1/200 working dilution. Discontinued by the company). The mouse monoclonal anti-AR/Androgen Receptor Antibody (441), sc-7305 (Santa-Cruz Biotechnology) and rabbit monoclonal recombinant anti-Androgen Receptor antibody [EPR1535(2)] (ab133273, Abcam) were unsuccessful in staining human cryosections. For activated microglia/macrophages the mouse monoclonal anti-human CD68 antibody, clone PG-M1 (Dako, M0876; working dilution 1/100) was used. The following day, ready-to-use

secondary antibodies (ImmPRESS® Horse Anti-Rabbit IgG Polymer Kit, Peroxidase MP-7401 and horse anti-mouse IgG ImmPRESS® Secondary Antibody [AP Polymer], MP-5402, Vector Laboratories) were incubated at room temperature for 1 hr. Staining was developed with a DAB Peroxidase (HRP) Substrate Kit (Vector, SK-4100) and a VECTOR Blue AP Substrate Kit (Vector, SK-5300) as per manufacturer's guidelines. All sections were mounted using Mowiol mounting medium (475,904, MERCK- Millipore).

RT-qPCR analysis. Total RNA from DHT-treated EAE female spinal cord and vehicle-treated EAE female spinal cord was isolated as described for bulk RNA-sequencing (main text) by using the Trizol Technique (Thermo Fisher Scientific) and RNeasy Mini Kit (Qiagen). Reverse Transcription was performed using the High-Capacity cDNA Reverse Transcription kit (Applied Biosystems). Quantitative real-time PCR was carried out by using the TaqMan Gene expression Master Mix (Thermo Fisher Scientific) and gene expression was analyzed with the 7300 Systems SDS Software (Applied Biosystems) normalized to reference genes GAPDH. TaqMan probes were as follows: GAPDH, Mm99999915_m1; Tnf, Mm00443258_m1; Csf2, Mm01290062_m1.

Bulk RNA-Seq deconvolution. We used CIBERSORTx tool[2] on the docker module Cibertsortx/fractions, with 100 permutations as input parameter, in order to deconvolute our bulk RNA-Seq datasets obtained from EAE spinal cord samples. The signature of scRNA-Seq matrix was generated according to the book methods described by Steen et al.[3] with the GSE113973 public scRNA-Seq dataset from mouse EAE model. The deconvolution analysis was performed on two mixture files corresponding to the RNA-Seq count matrices generated as described before, containing females and males' comparisons, DHTFvsCTF and DHTMvsCTM, respectively. The results obtained are estimated as the proportions of each cell types in each RNA-Seq sample inferred from the prior knowledge of the scRNA-Seq sample. R script has been deposited in https://github.com/ParrasLab/Androgen-signaling-and-remyelination-Nat-Commun-paper.

Statistical analysis. Statistical analysis of mouse histological staining was performed with GraphPad Prism 7.0 software (La Jolla, CA). The significance of differences between means was evaluated by the two-tailed Student's unpaired t test for two independent group comparisons when distribution was normal. In case of absence of normal distribution (analyzed D'Agostino & Pearson normality test and Shapiro-Wilk normality test), the non-parametric two-tailed Mann-Whitney test was used. Appropriate corrections were done according to the determination of the variance of each sample. The values are the means ± SEM from the number of animals indicated in each plotted graph or as indicated in the corresponding legends. Significance of $p<0.05$ was used for all analyses. *, $p\leq0.05$; **, $p\leq0.01$; ***, $p\leq0.001$; ****, $p<0.0001$. For transcriptomic analyses, multiple testing correction aimed at controlling the false discovery rate (FDR) was performed using the Benjamini-Hochberg method in supplementary tables 8 and 12. Cutoff used for FDR was 5%. In supplementary tables 3, 9, 10, 11, the workflow used edgeR's quasi-likelihood (QL) pipeline (edgeR-quasi) for differential expression. This statistical methodology uses negative binomial generalized linear models but with F-tests instead of likelihood ratio tests. This method provides stricter error rate control than other negative binomial based pipelines, including the traditional edgeR pipelines or DESeq2[4].

Therefore, we have used QL F-tests instead of the more usual likelihood ratio tests (LRT) as they give stricter error rate control by accounting for the uncertainty in dispersion estimation, using the glmQLFTest function. In addition, in order to control the false discovery rate (FDR), multiple testing correction is performed using the Benjamini-Hochberg method. The total number of DEGs (differentially expressed genes) were identified at an FDR of 5% with decideTestsDGE function.

Supplementary references

1. Macnair W*, et al.* Single nuclei RNAseq stratifies multiple sclerosis patients into three distinct white matter glia responses. *bioRxiv 20220406487263*, (2022).

2. Newman AM*, et al.* Determining cell type abundance and expression from bulk tissues with digital cytometry. *Nat Biotechnol* **37**, 773-782 (2019).

3. Steen CB, Liu CL, Alizadeh AA, Newman AM. Profiling Cell Type Abundance and Expression in Bulk Tissues with CIBERSORTx. *Methods Mol Biol* **2117**, 135-157 (2020).

4. Chen Y, Lun A, Smyth G. From reads to genes to pathways: differential expression analysis of RNA-Seq experiments using Rsubread and the edgeR quasi-likelihood pipeline [version 2; peer review: 5 approved]. *F1000Research* **5**, (2016).
