# Supplementary material for: Androgens show sex-dependent differences in myelination in immune and non-immune murine models of CNS demyelination
Source: Nat Commun. 2023 Mar 22;14:1592. doi: 10.1038/s41467-023-36846-w (PMC10033728; doi:10.1038/s41467-023-36846-w)
Supplement: Supplementary file 3 — Description of Additional Supplementary Files [file 41467_2023_36846_MOESM3_ESM.pdf]

Description of Additional Supplementary Files

File Name: Supplementary Dataset 1:

Description : DEGs_females-males_cpm_EdgeR_FDR005.

File Name: Supplementary Dataset 2:

Description : Oligodendrogenesis scoring_all genes_females.

File Name: Supplementary Dataset 3:

Description : Oligodendrogenesis scoring_all genes_males.

File Name: Supplementary Dataset 4:

Description : Oligodendrogenesis scoring_DEGs_females.

File Name: Supplementary Dataset 5:

Description : Oligodendrogenesis scoring_DEGs_males.

File Name: Supplementary Dataset 6:

Description : GO_DHT_females-males_EdgeR_FDR005.

File Name: Supplementary Dataset 7:

Description : Microglia_genes.

File Name: Supplementary Dataset 8:

Description : Astroglia_genes.

File Name: Supplementary Dataset 9 :

Description : DEGs-microglia_astroglia_females-males.

File Name: Supplementary Dataset 10:

Description : GSEA_microglial_genes.

File Name: Supplementary Dataset 11:

Description : Deconvolution_bulk_RNA-Seq.
